# C4d Immunoreactivity in Autoimmune and HBV-Induced Hepatitis: Implications for Complement-Mediated Hepatocellular Injury

**DOI:** 10.3390/pathophysiology32030030

**Published:** 2025-07-01

**Authors:** Ye Zheng, Haitao Tong, Wenjuan Guo, Ao Wang, Wenxing Hu, Min Wu, Xiaonan Zhang

**Affiliations:** 1 Shanghai Public Health Clinical Center, Fudan University, Shanghai 200433, China; zhengye@shaphc.org (Y.Z.); tonghaitao@shaphc.org (H.T.); guowenjuan@shaphc.org (W.G.); wangao@shaphc.org (A.W.); wummi1981@163.com (M.W.); 2 Faculty of Science and Technology, University of Canberra, Canberra, ACT 2617, Australia

**Keywords:** immune complex, chronic hepatitis B, CHB, autoimmune hepatitis, AIH, C4d deposition

## Abstract

Background: Although immune complex formation is widely acknowledged as the etiological agent for the development of systemic lupus erythematosus, polyarteritis nodosa, reactive arthritis, etc., its roles in chronic hepatitis are less understood. This study aims to compare the immunohistochemistry profile of immune complex deposition in patients with chronic hepatitis B (CHB) and autoimmune hepatitis (AIH). Methods: Immunohistochemistry of C4d, a widely used marker for complement deposition was employed on liver biopsies from 72 and 15 patients with CHB and AIH, respectively. Statistical analysis was performed to analyze its prevalence and its association with a range of clinical and histological parameters. Results: Among the 15 AIH biopsies examined, C4d deposition was observed in 11 cases (73.3%), the majority of which showed a periportal staining pattern (10/11). In CHB, 61 (84.7%) of 72 cases tested positive for C4d, which did not differ significantly with that of AIH. While the periportal pattern was predominantly observed in CHB cases, positive staining in central veins, sinusoids, and hepatic parenchyma were also documented. In particular, C4d deposition is significantly associated with elevated serum ALT and liver inflammation in CHB. Of note, in specimens with a patchy parenchymal C4d staining pattern, a spatially correlated HBsAg IHC signal was observed in adjacent sections from the same tissue. Conclusions: These data suggest an involvement of immune complex-mediated immunopathy in autoimmune hepatitis and HBV-induced hepatitis. The positive intrahepatic C4d signal was associated with heightened liver inflammation. The colocalization of the C4d signal on hepatocytes with HBsAg strongly suggests a causal relationship between viral activity and complement deposition. These observations align with our recent evidence implicating the contribution of capsid–antibody complexes in the pathogenesis of CHB.

## 1. Introduction

The humoral immune response serves as an essential arm of the immune system against various forms of insults. However, it may also trigger pathologies when the target antigens are persistently present in circulation or on the cell surface as a result of type II (antibody-mediated) and type III (immune complex-mediated) hypersensitivity. A typical example is systemic lupus erythematosus (SLE) in which immune complexes form in the glomeruli and capillaries leading to kidney (glomerulonephritis) and blood vessel (necrotizing vasculitis) pathologies [1].

Liver, as an essential organ of metabolism but also gradually appreciated as an immune organ, is a target for a series of inflammatory diseases, including autoimmune hepatitis (AIH), viral hepatitis, alcoholic hepatitis, drug-induced hepatitis, etc. AIH is a complex autoimmune disease characterized by positive circulating autoantibodies, elevated serum IgG and transaminase levels, a strong female predominance, and the presence of interface hepatitis in liver histology [2,3]. Due to the presence of autoantibodies and high IgG levels, the formation of antibody–antigen complexes and the activation of a complement system in the liver can be expected [3,4], but inadequate pathological investigation has been undertaken. In addition, viral hepatitis is an important group of inflammatory liver diseases, among which hepatitis B virus (HBV) and hepatitis C virus (HCV) are the most prevalent. In chronic hepatitis C, viral particles and core proteins induce a large amount of IgM with rheumatoid factor activity which forms cold-precipitable, multimolecular immune complexes with viral particles called cryoglobulins leading to cryoglobulinemic vasculitis [5]. In chronic hepatitis B (CHB), the HBV surface antigen immune complex can lead to polyarteritis and membranous glomerulonephritis but is not directly linked to liver histopathology [6]. Nevertheless, there have been reports suggesting a possible role of HBV capsid–antibody complexes (CACs) in mediating chronic liver inflammation [7,8,9]. This necessitates further study on the histological evidence of complement activation in CHB and its relationship with key clinical and virological parameters.

Complement component 4d (C4d) is a product of complement C4 cleavage, mainly involved in the activation of the classical pathway, during which C4d is attached to the vascular endothelium, basement membrane, or cell surface membrane through covalent bonds. Therefore, C4d is a specific, enduring marker of antibody-mediated immune response. C4d deposition, first described in kidney allografts [10,11] and later also observed in other transplanted organs [12], is used to determine the humoral component of rejection. C4d staining is now considered as one of the criteria for diagnosing acute antibody-mediated rejection after liver transplant [13,14]. In addition, there have been studies on the use of C4d deposition for diagnosing and monitoring autoimmune and infectious diseases, including systemic lupus erythematosus (SLE) and lupus nephritis (LN) and coronavirus infection [15,16,17].

In this study, we aimed to evaluate the level of complement activation using immunohistochemistry of C4d in liver biopsies from adult patients with AIH and CHB. Furthermore, its relationship with various virological, immunological, and histological characteristics in CHB patients was further analyzed.

## 2. Materials and Methods

### 2.1. Patients and Specimens

We performed a retrospective analysis of 87 hospitalized patients in Shanghai Public Health Clinical Center, Fudan University, among whom 15 patients had a diagnosis of AIH and 72 had CHB. The diagnostic criteria of AIH include the measurement of autoantibody (titers of antinuclear antibodies (ANAs) and anti-smooth muscle antibodies (ASMAs), immunoglobulin G levels (serum concentrations of IgG above normal), the evaluation of liver histology (evidence of interface hepatitis, lymphoplasmacytic infiltrate, and rosette formation), and the exclusion of viral hepatitis including HAV, HBV, and HCV. A score of 6 is considered as probable AIH and a score of ≥7 as definite AIH [18]. The diagnosis of CHB was based on serological and virological results for HBV. Liver tissue samples were obtained by percutaneous biopsy. The biopsy specimen was fixed with 10% neutral formalin, dehydrated, embedded in paraffin block, sliced with a thickness of 3 μm, and then dried at 65 °C for 2 h.

### 2.2. Immunohistochemistry (IHC)

Immunostaining was carried out on a Leica BOND-Max (Leica Biosystems, Nussloch, Germany) automatic IHC system. Briefly, after Bond dewax solution and alcohol dewaxing, antigen retrieval, endogenous peroxidase activity was quenched by Peroxide Block. The staining steps involved a 25 min primary antibody anti-C4d (ZA-0564, clone: SP91, ZSGB-BIO, Beijing, China) incubation, an 8 min post-primary incubation, an 8 min polymer incubation, an 8 min DAB incubation, and a 10 min hematoxylin incubation. Slides were then dehydrated, cleared, cover-slipped, and scored by pathologists.

### 2.3. Pathologic Evaluation

All the IHC results were evaluated independently by two experienced practicing pathologists. Positive C4d staining is defined as significant granular or punctate staining in the portal area, central veins, sinusoids, or parenchyma. A slide with two or more positive fields is considered a positive result. The grading and staging for inflammation and fibrosis in the liver was performed according to the Scheuer system [19] with a four-category scale.

### 2.4. Statistics

GraphPad Prism 9.0 was used for data statistical analysis. The Fisher’s exact test was used for count data. For measurement data, the normality and lognormality tests were firstly performed. Then, the unpaired *t* test was used for two groups of continuous data, and the Mann–Whitney U test was used for two groups of non-parametric continuous data. *p* value < 0.05 was considered statistically significant.

## 3. Results

Fifteen cases of AIH and seventy-two cases of CHB, from whom liver biopsies were collected, were included in this study. The baseline characteristics are summarized in Table 1. In AIH patients, the median age at diagnosis was 57 years, with 14 cases being female (93.9%), and the median serum ALT level was 123 U/L. In CHB patients, the median age at diagnosis was 41 years, with a comparable male to female ratio (47.2%:52.8%), and the median serum ALT level was 38 U/L. C4d was positive in 11 (73.3%) of 15 AIH biopsies and in 61 (84.7%) of 72 CHB biopsies (Table 1). No significant difference in the rate of positive staining for Cd4 was observed between biopsies from AIH and from CHB patients (*p* = 0.28, Fisher’s exact test).

**Figure 1:**
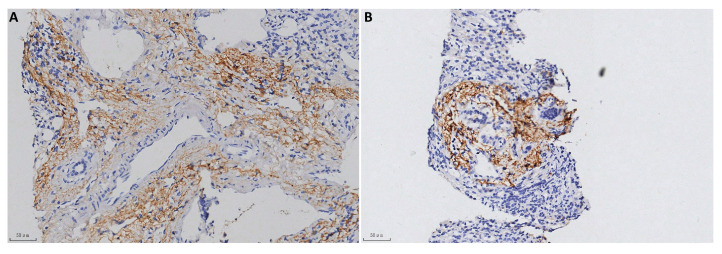
C4d immunostaining in AIH (100×). (**A**) Strong C4d immunostaining in the portal tracts. (**B**) Lymphoid aggregate accentuation.

**Figure 2:**
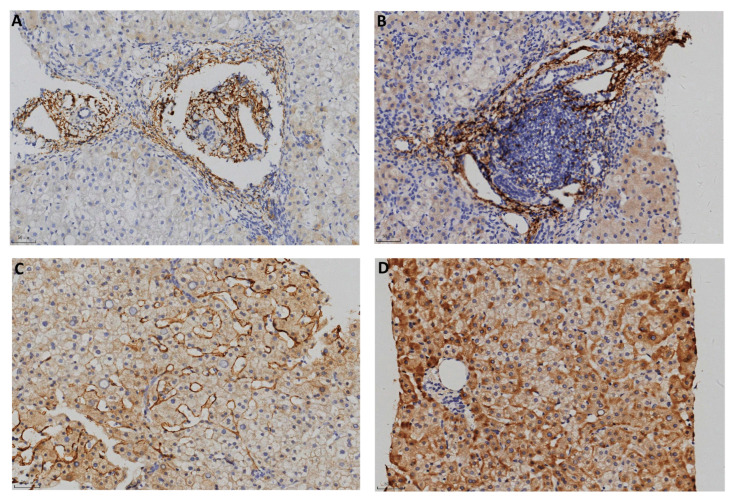
C4d immunostaining in CHB (100×). (**A**) Portal tracts. (**B**) Portal area with lymphoid aggregation. (**C**) Sinusoids. (**D**) Hepatic parenchyma.

The typical positive C4d immunostaining patterns in each group are shown in Figure 1 and Figure 2 and negative staining results were shown in Supplementary Figure S1. In the AIH cases, 10 positive biopsies showed portal tract deposition. In a typical AIH case, C4d was strongly deposited in the portal veins, capillaries, and periportal sinusoidal endothelium involving most of the portal tracts (Figure 1A). Lymphoid aggregate accentuation of C4d staining was also observed in an AIH biopsy (Figure 1B). In CHB, C4d deposition exhibited more diverse patterns. In addition to portal tract deposition (Figure 2A,B), central vein staining (4 cases, 5.6%) and sinusoid staining (11 cases, 15.3%) was also observed (Figure 2C). Strong staining of hepatic parenchyma was observed in nine cases (12.5%, Figure 2D).

**Figure 3:**
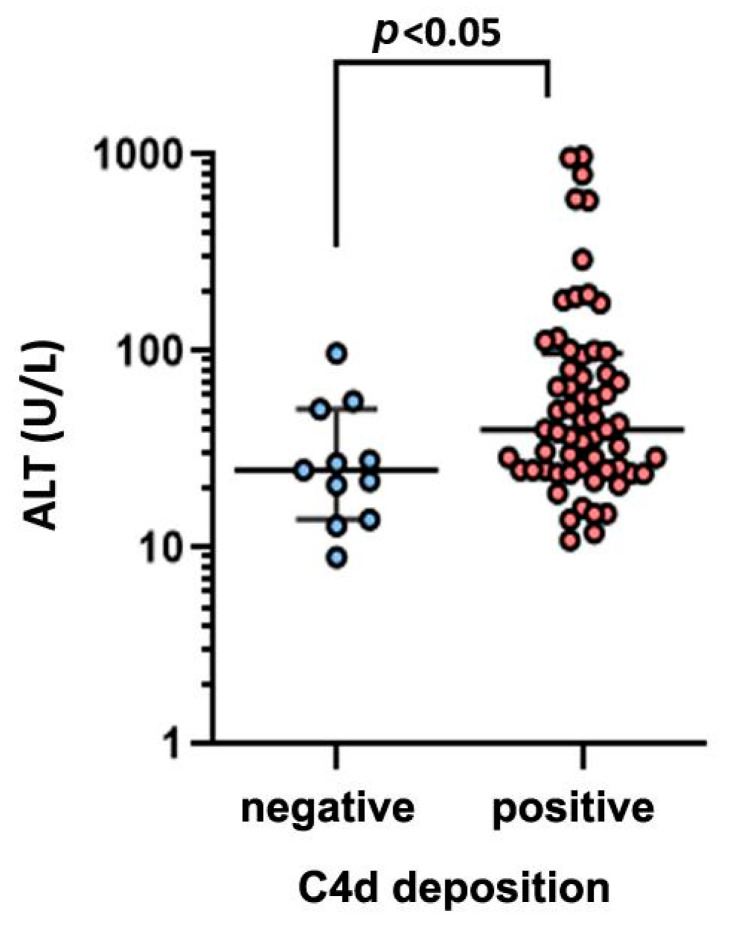
Comparison of serum ALT level between C4d-negative and -positive groups in CHB patients.

**Figure 4:**
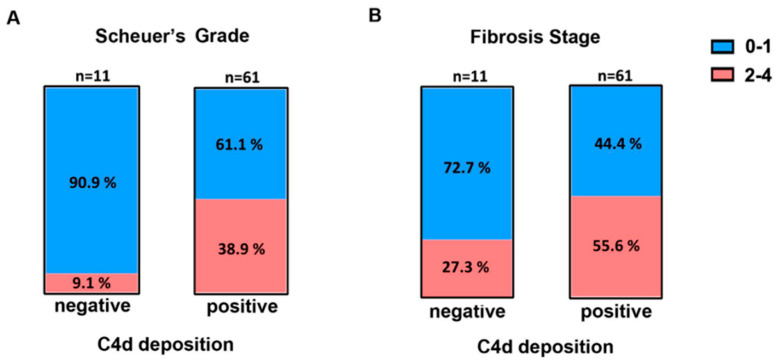
Frequency of Scheuer’s liver grade (**A**) and fibrosis stage (**B**) in C4d-negative and -positive groups in CHB patients. Blue: Grades or stages 0–1, Red: grades or stages 2–4.3.

Patients with CHB go through four major clinical stages (immune-tolerant, immune-active, carrier, and e-negative hepatitis) with distinct virological, immunological, and histological characteristics. We first analyzed the relationship between C4d deposition and serum ALT, HBsAg, HBeAg, HBcAb, and HBV DNA (Table 2). The serum ALT level in C4d-positive patients was significantly elevated compared with that in C4d-negative patients (C4d-positive median = 40 IU/L vs. C4d-negative median = 25 IU/L, *p* < 0.05 Figure 3). We then analyzed the relationship between C4d deposition and liver histological data. Using Scheuer’s liver histological grading (inflammation) scheme, we categorized patients into two groups: G0–1 with minimal inflammation and G2–3 with moderate or severe inflammation. The proportion of G2–3 grading in C4d-positive patients was significantly elevated compared with that in C4d-negative patients (Figure 4A, *p* < 0.05, Fisher’s exact test). Likewise, we categorized patients based on the staging as S0-1 for mild fibrosis and S2–S4 for moderate to severe fibrosis. No significant difference in S grading was observed between C4d-positive and -negative patients (*p* = 0.32, Figure 4B). These results suggest a link between complement deposition and intrahepatic inflammation.

**Figure 5:**
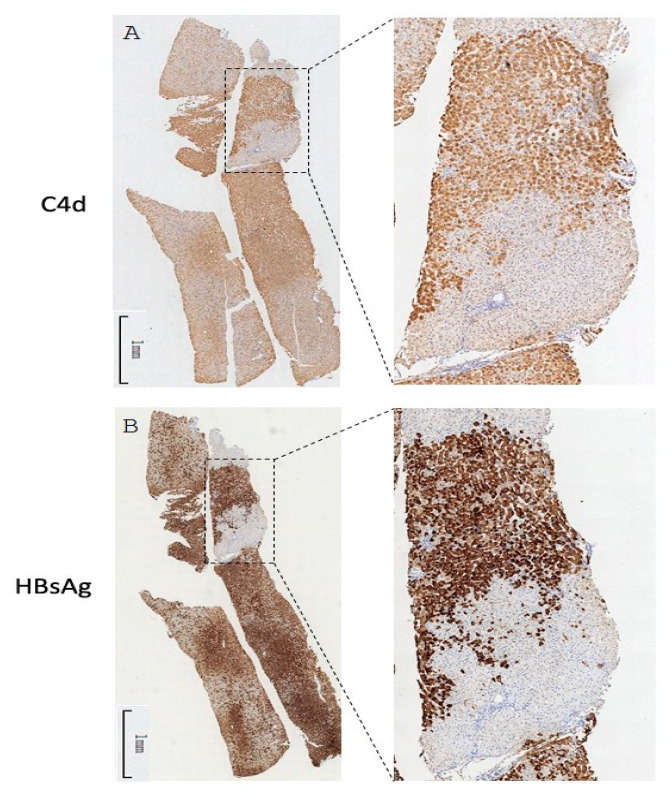
Whole-tissue scan of C4d (**A**) and HBsAg (**B**) IHC from one CHB liver specimen. One pair of representative images is shown. The selected regions were magnified in the inset.

Our previous report proposed the existence of a virus-specific immune complex and its role in inducing chronic hepatitis [7]. We thus explored whether or not the observed complement deposition was spatially correlated with viral activity. Although only a minority of cases showed C4d signal on parenchymal cells, some of them exhibited a clustered pattern reminiscent of the typical HBsAg staining results [20]. We therefore compared the C4d and HBsAg IHC results generated from adjacent sections of the same tissue blocks. As shown in Figure 5, the staining pattern of C4d and that of HBsAg showed intriguing overlaps with very similar positive clusters in identical liver lobules. A similar colocalization pattern was also found in three other cases). These results strongly suggest a linkage between active viral antigen expression and the intrahepatic activation of complement in CHB.

## 4. Discussion and Conclusions

The complement system is a highly conserved and an essential component of the innate immune system and works synergistically with the adaptive immunity. Meanwhile, the liver is an important immune organ where most complement proteins are produced and primary hematopoietic cells originate from during fetal development. A diverse array of liver inflammatory diseases—arising from infections, environmental factors, lifestyle choices, or genetic predisposition—may involve the complement system in their pathogenesis. Unfortunately, very few pathological studies were performed in this regard.

Autoimmune hepatitis (AIH) is characterized by the presence of autoantibodies and high IgG levels; thus, the formation of antibody–antigen complexes and the activation of complement system in the liver seems plausible. However, the role and contribution of complement activation in autoimmune liver disease remains debated. Dorothée Bouron-Dal Soglio et al. reported an 83% (25 of 30) C4d positivity rate in pediatric AIH biopsies [4]. In another study on untreated adult AIH, C4d was positive in seven of the nine (77.8%) cases consistent with the pediatric study [3]. In our study, a 73.3% (11 of 15) positive rate was documented, which is comparable to these two reports. In our study, the staining of C4d in AIH biopsies was concentrated in the periportal area, where the bulk of the lymphocyte infiltration was located; hence, it is consistent with the “interface inflammation”, a key feature of AIH, and strongly associated with the pathogenesis of the disease.

Chronic hepatitis B is a persistent viral infection spanning decades in the affected individuals and exhibits variable and complex disease patterns. Persistent infection is usually the result of vertical transmission during an early stage of life, whose initial phase is characterized by high viral replication and antigen expression concomitant with a largely tolerized virus-specific immunity. This is followed by the next phase, typically when the infected individual reaches adulthood, with varying degrees of liver inflammation accompanied by a fluctuating decrease in viral activity. During this process, chronic liver disease slowly progresses with liver fibrosis, cirrhosis, and hepatocellular carcinoma. There has long been a focus on the cell-mediated immune response in the pathogenesis of CHB where the roles of T cells, B cells, and NK cells were especially scrutinized [9,21]. However, the precise sequence of immunological processes culminating in this condition remains a subject of considerable debate. On the other hand, the contribution of humoral immunity in this process is poorly characterized. We previously identified a novel viral particle, dubbed CACs (capsid–antibody complexes), which is formed by the premature release of naked capsids into the circulation [7]. Furthermore, a simple ELISA assay was developed to quantify CACs in serum of CHB patients. CACs were found to be highly prevalent in CHB patients (>70% positive rate), and their levels closely correlate with liver inflammation and outperform serum ALT levels in revealing cryptic liver injury [8]. As CACs are in essence immune complexes capable of eliciting complement activation and downstream events, we therefore evaluated the level of complement deposition in the liver of CHB patients using the immunohistochemistry of C4d. We observed an 84.7% (61/72) positive rate of C4d in hepatitis B biopsies. In another study focusing on pediatric hepatitis B, C4d was reported to be positive in 17 (89%) of 19 hepatitis B biopsies, and 6 (40%) of 15 hepatitis C biopsies [4]. The pattern of C4d staining in CHB biopsies was slightly different from that in AIH. In addition to the periportal staining pattern, a small portion of C4d signal was observed in central veins (5.6%), sinusoids (15.3%), and hepatic parenchyma (12.5%). By contrast, central vein C4d staining was observed in 58% of pediatric CHB cases [4].

Despite the high prevalence of the C4d signal in CHB, its relationship with various virological, immunological, and histological characteristics has not been explored. Bugdaci et al. reported the inverse correlation of serum C4 levels with serum ALT levels and the child score in patients with CHB [22], suggesting the consumption of complement proteins due to the presence of immune complexes. We found in our CHB cohort that C4d deposition is associated with elevated serum ALT and liver inflammation reconfirming our previous findings. Moreover, an inspection of C4d IHC sections with parenchymal pattern side-by-side with HBsAg IHC results revealed an intriguing spatial overlap. This is direct evidence supporting the causative role of viral activity in eliciting the complement system. As anti-HBs is mostly absent in chronically infected individuals, the initiator for the complement attachment is most likely caused by other viral antigens such as capsids presented on cell membrane of hepatocytes [8].

**Figure 6:**
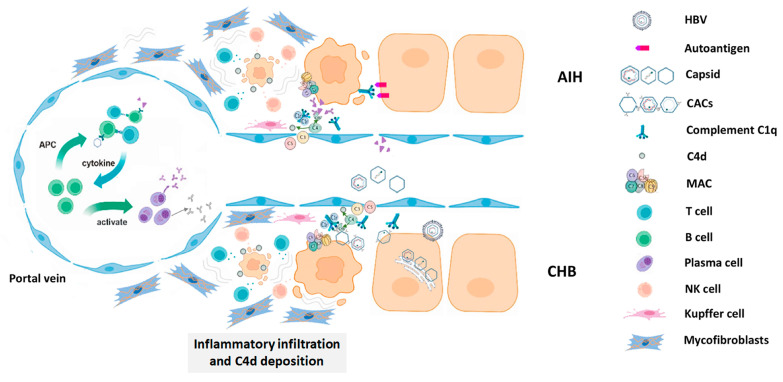
A schematic diagram of the possible mechanism of complement-mediated hepatocellular injury.

In conclusion, our results demonstrate an involvement of immune complex reaction in autoimmune hepatitis and chronic hepatitis B probably by eliciting an array of subsequent immunological events as shown in Figure 6. Although C4d immunohistochemistry is not specific for AIH or CHB, these data confirm the involvement of the immune complex in the pathogenesis of chronic hepatitis B and suggest the possible causative role of capsid–antibody complexes (CACs) in the process [23].

## Figures and Tables

**Table 1:** Summary of clinical characteristics of the patients and C4d results.

Inflammatory Liver Disease	Autoimmune Hepatitis	Chronic Hepatitis B
No. of biopsies	15	72
Gender (Male/Female)	1:14	34:38
Age (years) *	57 (56, 64)	41 (33, 49.75)
ALT (IU/L) *	123 (35, 137)	38 (24.25, 80)
C4d-positive biopsies n (%)	11 (73.3)	61 (84.7)
Portal area n (%)	10 (66.7)	58 (80.6)
Centrolobular vein n (%)	1 (0.07)	4 (5.6)
Hepatic sinusoid n (%)	2 (0.13)	11 (15.3)
Hepatic parenchyma n (%)	0	9 (12.5)

* Data are expressed as median (1st–3rd quartiles).

**Table 2:** Summary of the key virological and serological variables and C4d results.

Chronic Hepatitis B	C4d Deposition		*p* Value
	Negative	Postive	
No. of biopsies	11	61	
Gender (Male/Female)	3:8	31:30	0.35 ^1^
Age (years)	38 (33, 47)	41 (33, 50)	0.70 ^2^
ALT (IU/L) *	25 (14, 51)	40 (25, 97.5)	0.02 ^3^
Serum HBsAg (log_10_ IU/mL)	3.66 (2.19, 4.17)	3.55 (2.98, 3.96)	0.87 ^3^
Serum HBeAg (S/CO)	0.56 (0.42, 0.62)	0.67 (0.42, 473.8)	0.35 ^3^
Serum HBcAb (S/CO)	8.51 (8.17, 8.8)	8.8 (8.24, 9.31)	0.17 ^3^
Serum HBV DNA (log_10_ IU/mL)	3.74 (2.84, 6.71)	4.76 (2.86, 6.91)	0.42 ^3^

Data are expressed as median (1st–3rd quartiles) except gender. * *p* < 0.05. 1: Fisher’s exact test; 2: Unpaired *t* test; 3: Mann–Whitney U test.

## Data Availability

The underlying data and the images will be available upon reasonable request.

## Supplementary Materials

The following supporting information can be downloaded at: https://www.mdpi.com/article/10.3390/pathophysiology32030030/s1, Figure S1: Negative result for immunohistochemistry of C4d from the liver biopsy sections.
