# Supplementary figures and images for: C4d Immunoreactivity in Autoimmune and HBV-Induced Hepatitis: Implications for Complement-Mediated Hepatocellular Injury
Source: Pathophysiology. 2025 Jul 1;32(3):30. doi: 10.3390/pathophysiology32030030 (PMC12285942; doi:10.3390/pathophysiology32030030)

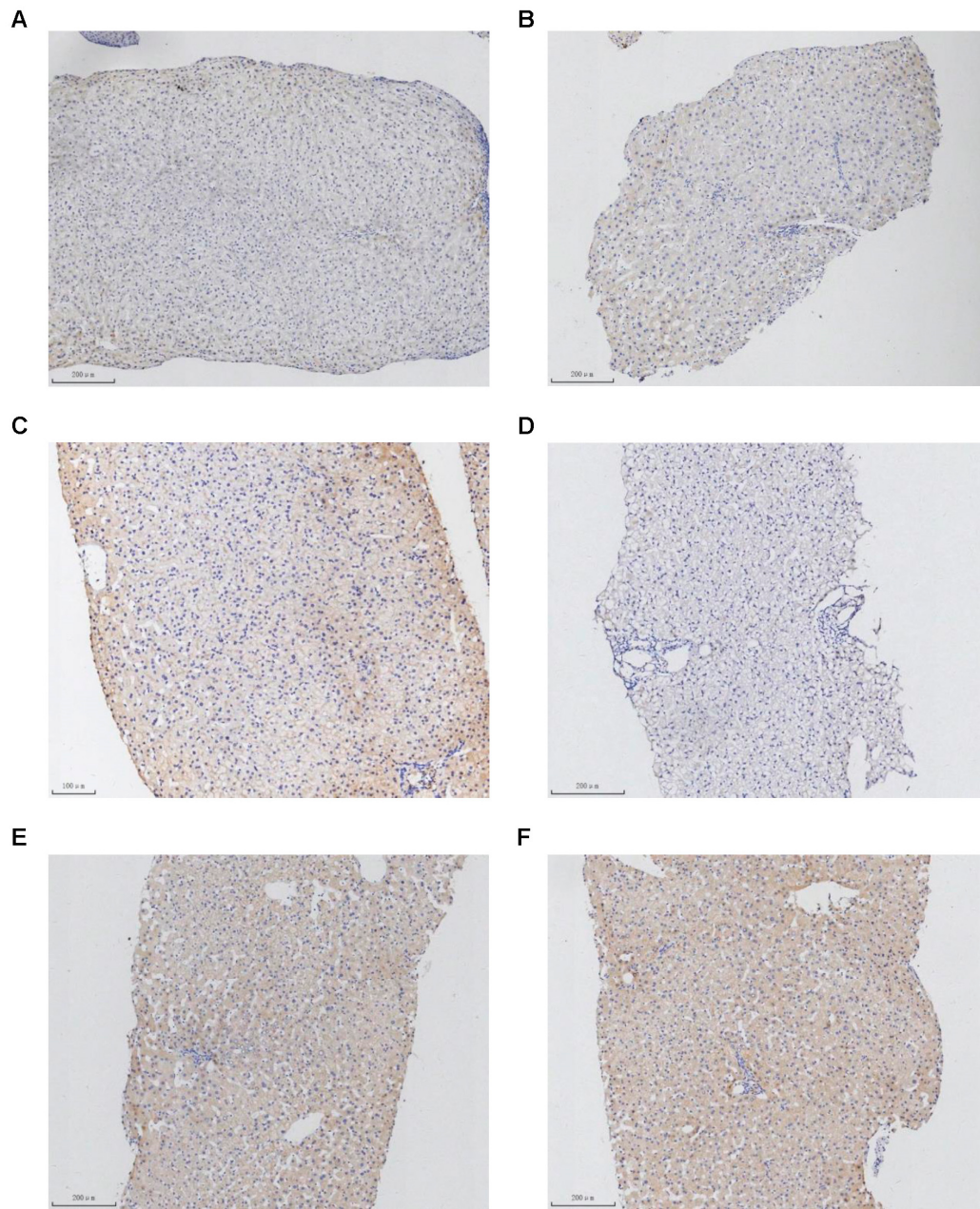

**Figure S1.** Negative result for immunohistochemistry of C4d from the liver biopsy sections.

Supplement: Supplementary file 1 [file pathophysiology-32-00030-s001.zip › pathophysiology-3548139-supplementary.pdf]
